# Corrigendum to “On a Nonlinear Wave Equation Associated with Dirichlet Conditions: Solvability and Asymptotic Expansion of Solutions in Many Small Parameters”

**DOI:** 10.1155/2017/9841725

**Published:** 2017-11-05

**Authors:** Le Thi Phuong Ngoc, Le Khanh Luan, Tran Minh Thuyet, Nguyen Thanh Long

**Affiliations:** ^1^Nhatrang Educational College, 01 Nguyen Chanh Street, Nhatrang City, Vietnam; ^2^Department of Mathematics, University of Economics of Ho Chi Minh City, 59C Nguyen Dinh Chieu Street, District 3, Ho Chi Minh City, Vietnam; ^3^Department of Mathematics and Computer Science, University of Natural Science, Vietnam National University Ho Chi Minh City, 227 Nguyen Van Cu Street, District 5, Ho Chi Minh City, Vietnam

In the article titled “On a Nonlinear Wave Equation Associated with Dirichlet Conditions: Solvability and Asymptotic Expansion of Solutions in Many Small Parameters” [1], there are similarities with two of the authors' previous publications; one of them was cited:

1. Le Thi Phuong Ngoc, Le Khanh Luan, and Nguyen Thanh Long, “An asymptotic expansion of a weak solution for a nonlinear wave equation,” Acta Mathematica Vietnamica, Volume 36, Number 3, 2011, pp. 695–722.
2. Le Thi Phuong Ngoc, Le Khanh Luan, Tran Minh Thuyet, and Nguyen Thanh Long, “On the nonlinear wave equation with the mixed nonhomogeneous conditions: linear approximation and asymptotic expansion of solutions,” Nonlinear Analysis, 71 (2009) 5799–5819 (cited as reference [14]).

This article [1] cited the earlier article [2]; however, articles [1, 3] did not cite each other as article [1] was submitted later and accepted earlier.

In this article [1], we considered the following Dirichlet problem:

(1) utt−∂∂xμx,t,uux=fx,t,u,ux,ut,0<x<1,  0<t<T,u0,t=u1,t=0,ux,0=u~0x,utx,0=u~1x.

Using the Faedo-Galerkin method and the compact imbedding theorems, first, we proved the solvability and the uniqueness of a weak solution of problem (1). Based on the ideals and the techniques used in the mentioned papers, we studied a high-order asymptotic expansion of a weak solution for problem (1), where (1)_1_ has the form of a linear wave equation with nonlinear perturbations containing many small parameters *ε*_1_,…, *ε*_*p*_ as follows:

(2) utt−∂∂xμ0x,t+∑i=1pεiμix,t,uux=f0x,t+∑i=1pεifix,t,u,ux,ut,0<x<1,  0<t<T,u0,t=u1,t=0,ux,0=u~0x,utx,0=u~1x.

In article [3], we studied the following problem:

(3) utt−∂∂xμuux=fx,t,u,ux,ut,0<x<1,  0<t<T,u0,t=u1,t=0,ux,0=u~0x,utx,0=u~1x.

Equation (3)_1_ constitutes a relatively simple case of (1)_1_, that is, *μ*(*x*, *t*, *u*) = *μ*(*u*). Also, applying the Faedo-Galerkin method and the compact imbedding theorems, we proved the existence of a unique weak solution of (3). The main result is an asymptotic expansion of high order in many small parameters *ε*_1_,…, *ε*_*p*_ of the weak solution *u* for the problem

(4) utt−∂∂xμ0u+∑i=1pεiμiuux=fx,t,u,ux,ut+∑i=1pεifix,t,u,ux,ut,0<x<1,  0<t<T,u0,t=u1,t=0,ux,0=u~0x,utx,0=u~1x.

In the earlier article [2], we studied the following problem:

(5) utt−∂∂xμuux=fx,t,u,ux,ut,0<x<1,  0<t<T,ux0,t=gt,u1,t=0,ux,0=u~0x,utx,0=u~1x.

The boundary conditions in this problem and in the above problem are different. Applying the Faedo-Galerkin method and the compact imbedding theorems, we proved the existence of a unique weak solution of (5). We established an asymptotic expansion of high order in two small parameters *ε*_1_, *ε*_2_ of the weak solution *u* for the problem

(6) utt−∂∂xμ0u+ε1μ1uux=fx,t,u,ux,ut+ε2f1x,t,u,ux,ut,0<x<1,  0<t<T,ux0,t=gt,u1,t=0,ux,0=u~0x,utx,0=u~1x.

The novelty that each of the three articles introduces is shown below.

First, the problems considered are different because the boundary conditions in (5), *u*_*x*_(0, *t*) = *g*(*t*) and *u*(1, *t*) = 0, and in (1) and (3), *u*(0, *t*) = *u*(1, *t*) = 0, are different. Although (1) and (3) are the same, (2) and (4) are different.

Second, we applied the Faedo-Galerkin method and the compact imbedding theorems to prove the existence, but the techniques used in proofs and in estimates for each problem are different. We have to choose the spaces and inequalities with respect to the problem. On the other hand, with (5), we need the transformation *u*(*x*, *t*) ↦ *v*(*x*, *t*) = *u*(*x*, *t*)−(*x* − 1)*g*(*t*) to reformulate problem given as a problem with homogeneous boundary conditions.

Third, the ideals in establishment of asymptotic expansions for each problem are different.

(7) 1  utt−∂∂xμ0x,t+∑i=1pεiμix,t,uux=f0x,t+∑i=1pεifix,t,u,ux,ut;2  utt−∂∂xμ0u+∑i=1pεiμiuux=fx,t,u,ux,ut+∑i=1pεifix,t,u,ux,ut;3  utt−∂∂xμ0u+ε1μ1uux=fx,t,u,ux,ut+ε2f1x,t,u,ux,ut.

We constructed the perturbations problems (2), (4), and (6), which are not the same. One difference is, in articles [2, 3], the term *u*_0_ with respect to *γ*_1_ = ⋯ = *γ*_*p*_ = 0 defined from the nonlinear problem. But in this article [1], in the high-order asymptotic expansion for the weak solution *u* of (2),

(8) u=∑γ1+⋯+γp≤Nuγ1⋯γpε1γ1⋯εpγp+Oε12+⋯+εp2N+1≈∑γ1+⋯+γp≤Nuγ1⋯γpε1γ1⋯εpγp.

The terms *u*_*γ*_1_⋯*γ*_*p*__  (*γ*_1_ + ⋯+*γ*_*p*_ ≤ *N*) are found in the linear problems, respectively, because *u*_0_ in (8) is the weak solution of the linear problem

(9) utt−∂∂xμ0x,tux=f0x,t,0<x<1,  0<t<T,u0,t=u1,t=0,ux,0=u~0x,utx,0=u~1x.
